# Analysis of post-operative changes in serum protein expression profiles from colorectal cancer patients by MALDI-TOF mass spectrometry: a pilot methodological study

**DOI:** 10.1186/1477-7819-8-33

**Published:** 2010-04-26

**Authors:** Christopher CL Liao, Anuja Mehta, Nicholas J Ward, Simon Marsh, Tan Arulampalam, John D Norton

**Affiliations:** 1 ICENI Centre, Department of Surgery, Colchester Hospital University NHS Foundation Trust, Turner Road, Colchester, CO4 5JL UK; 2 Department of Biological Sciences, University of Essex, Wivenhoe Park, Colchester, CO4 3SQ, UK

## Abstract

**Background:**

Mass spectrometry-based protein expression profiling of blood sera can be used to discriminate colorectal cancer (CRC) patients from unaffected individuals. In a pilot methodological study, we have evaluated the changes in protein expression profiles of sera from CRC patients that occur following surgery to establish the potential of this approach for monitoring post-surgical response and possible early prediction of disease recurrence.

**Methods:**

In this initial pilot study, serum specimens from 11 cancer patients taken immediately prior to surgery and at approximately 6 weeks following surgery were analysed alongside 10 normal control sera by matrix-assisted laser desorption ionisation time of-flight-mass spectrometry (MALDI-TOF MS). Using a two-sided t-test the top 20 ranked protein peaks that discriminate normal from pre-operative sera were identified. These were used to classify post-operative sera by hierarchical clustering analysis (Spearman's Rank correlation) and, as an independent 'test' dataset, by *k*-nearest neighbour and weighted voting supervised learning algorithms.

**Results:**

Hierarchical cluster analysis classified post-operative sera from all six early Dukes' stage (A and B) patients as normal. The remaining five post-operative sera from more advanced Dukes' stages (C1 and C2) were classified as cancer. Analysis by supervised learning algorithms similarly grouped all advanced Dukes' stages as cancer, with four of the six post-operative sera from early Dukes' stages being classified as normal (*P *= 0.045; Fisher's exact test).

**Conclusions:**

The results of this pilot methodological study illustrate the proof-of-concept of using protein expression profiling of post-surgical blood sera from individual patients to monitor disease course. Further validation on a larger patient cohort and using an independent post-operative sera dataset would be required to evaluate the potential clinical relevance of this approach. Prospective data, including follow-up on patient survival, could in the future, then be evaluated to inform decisions on individualised treatment modalities.

## Background

Treatment options for colorectal cancer (CRC) patients are still largely reliant on traditional staging criteria [1]. For early stage (Dukes' A; Stage I) patients, surgery alone offers the best prospect for cure [2]. For patients with advanced disease (Dukes' C1, C2 and D; Stages III or IV) with lymph node involvement or distant metastasis, there are established survival benefits of adjuvant chemotherapy [3,4]. Controversy still exists however, in optimum treatment modality for the 30% of patients who present with Dukes' B/Stage II disease [5-7]. Although 25 - 30% of these patients will develop recurrent disease, the decision to implement adjuvant chemotherapy is often made on the basis of additional perceived risk factors in an attempt to minimise chemotherapy-related morbidity [8]. There is therefore a long-recognised need for more robust criteria in patient stratification of the heterogeneous group of Dukes' B/Stage II patients.

In recent years, molecular markers present in patients' tumour cells have become established as centre-stage in the quest for improved cancer sub-group classification with the ultimate aim of developing individualised therapeutic regimes [9]. In CRC, markers based on microsatellite instability (*MSI*) and loss of heterozygosity at 18q have shown prognostic significance [10-13]. Of more specific relevance, gene-expression profiles based on 23- and 7-gene signatures are reportedly predictive for high risk of recurrence in Stage II disease [14-16].

More latterly, attention has focussed on analysis of the blood serum proteome as a potential source of tumour markers for disease diagnosis. By using matrix-assisted laser desorption-ionisation time of-flight-mass spectrometry (MALDI-TOF MS) or surface-enhanced laser desorption-ionisation time-of-flight mass spectrometry (SELDI-TOF MS), peptide fragments representing diagnostic molecular signatures from several types of cancer have been identified in blood sera/plasma that discriminate cancer patients from normal individuals [17-20]. In CRC, the sensitivity and specificity of this approach reportedly ranges from 90 - 95% [14,17,19,21-24]. Importantly, analysis of the diagnostic 'peptidome' of patients' blood serum is amenable to repeated sampling and might potentially be used to monitor disease course following surgery with the aim of predicting disease recurrence.

We report here the results of an initial pilot methodological study in which MALDI-TOF MS analysis of blood sera from a cohort of 11 colorectal cancer patients was performed immediately prior to and at approximately 6 weeks post-surgery (the usual time for commencement of adjuvant chemotherapy). The post-operative serum peptidome profile of patients who initially presented with early stage disease and who would be expected to experience long-term disease-free survival, mostly reverted to a more normal pattern. By contrast, the post-operative serum profiles of patients presenting with more advanced disease retained features more characteristic of cancer patients.

## Methods

### Serum samples

10 ml samples of venous peripheral blood were obtained from 10 healthy volunteers and from 11 patients diagnosed with CRC one day before their scheduled surgery and at approximately six weeks following surgery. All specimens were obtained with informed consent in accordance with UK NHS Research Ethics procedures (Protocol reference MH 528). Blood samples were collected using a sterile 'vacutainer' and were allowed to coagulate at room temperature. After 3 hours, samples were centrifuged at 1752 × g for 5 minutes and the sera recovered and filtered through a 0.45 μm 'minisart' SRP (Sartorius) membrane filter prior to being stored at -80°C. Table 1 summarises the Dukes' and TNM staging of the 11 patients studied.

**Table 1:** Clinico-pathological features of patient specimens.

^**1**^**T**	^**2**^**PO**	Age	Gender	Dukes' stage	^**3**^**TNM stage**	Differentiation	Vascular invasion	^**4**^**LNs harvested**	^**4**^**LNs positive**
T1	PO1	84	F	B	pT4, pN0, pR0	Moderate	Absent	15	0

T2	PO2	82	M	A	pT2, pN0, pR0	Moderate	Absent	9	0

T3	PO3	77	M	B	pT3, pN0, pR0	Poor	Absent	6	0

T4	PO4	71	F	A	pT2, pN0, pR0	Moderate	Present	11	0

T5	PO5	79	M	C1	pT3, pN1, pR0	Poor	Absent	23	3

T6	PO6	79	M	C1	pT4, p N1, pR0	Poor	Absent	15	3

T7	PO7	62	F	C2	pT2, pN2, pR0	Moderate	Present	14	8

T8	PO8	65	F	C2	pT4, pN1, pRx	Well to Mod	Present	15	2

T9	PO9	74	M	B	pT4, pN0, pMx, pRx	Moderate	Absent	7	0

T10	PO10	69	M	C1	pT3, pN2, pR0	Moderate	Absent	12	5

T11	PO11	62	M	B	pT3, pN0, pR0	Moderate	Absent	32	0

^1^T = Pre-operative specimen; ^2 ^PO = Post-operative specimen (six weeks following surgery); ^3^TNM stage = Tumour-Node-Metastasis classification where p = pathological stage, T1 = tumour invades submucosa, T2 = tumour invades muscularis propria, T3 = tumour invades subserosa, T4 = tumour breaches serosa and invades adjacent organ, N0 = no lymph nodes involved, N1 = 1-3 lymph nodes involved, N4 = four or more lymph nodes involved, R0 = resected margin free of cancer, Rx = resected margin not assessed, Mx = metastisis not assessed; ^4^LNs = lymph nodes

### Serum protein processing

In addition to following a strict regimen for sample collection and storage to minimise pre-analytical factors (above), subsequent processing steps were standardised in order to minimise possible between-sample variation. Following a single cycle of thawing, serum protein purification was performed on all samples in parallel by reverse-phase hydrophobic interaction chromatography using 'C8 MB-HIC' magnetic beads (Bruker Daltonics, Germany) following the manufacturer's instructions except that protein was eluted with 20 ul of 50% acetonitrile (Fisher Scientific UK). Eluted protein (20 ul) was mixed with 20 μl of 20 mg/ml of matrix, 2', 4', 6' - trihydroxy-acetophenone monohydrite (Fluka) in 50% acetonitrile and adjusted to 200 mM diammonium citrate. Slow co-crystallization [25] was performed at 21°C on an orbital shaker (1000 rpm) for four hours. Recovered crystals were washed with water and then deposited on a stainless steel MALDI target plate (Bruker Daltonics, Germany).

### MALDI-TOF mass spectrometry

Spectra were acquired on all samples in parallel using a MALDI-TOF mass spectrometer (Reflex IV; Bruker Daltonics) with the following settings: ion source 1, 20 kV; ion source 2, 16.65 kV; lens voltage 9.5 kV; pulsed ion extraction, 200 ns. Ionisation was achieved by irradiation with a nitrogen laser (ë = 337 nm) operating at 25 Hz and 35% laser power. For matrix suppression, we used a high gating factor with signal suppression up to 1000 Da. Mass spectra were detected in linear positive mode. Detector gain was set at 1600 V, sample rate at 1.0 and electronic gain at 100 mV with real-time smoothing. Spectra were acquired in duplicate from a single protein sample from each specimen from 500 shots delivered as 5 × 100 pulses. All protein peaks with signal-to-noise (S/N) ratio >3 in the mass range 1 - 20 kDa were recorded with the use of the 'AutoXecute' tool of the '*flexAnalysis*' acquisition software (Version 2.0; Bruker Daltonics). Spectra were externally calibrated using neurotensin and somatostatin (Sigma-Aldrich) as peptide calibrants. and were normalised using total ion current to determine the relative intensities if spectral peaks.

### Data Processing and analysis

Aligned spectra were exported as ASCII files and were digitally processed by smoothing (low pass filter), adaptive background correction and high pass filter before peak matching by using a mixture of Gaussians optimised by model-based clustering [26,27]. These and subsequent analysis were implemented in the '*GenePattern*' (Broad Institute, MIT, USA) suite of software tools [28]. Using a two-sided t-test, the top 20 ranked protein peaks that discriminate normal from pre-operative CRC sera were identified from the 356 aligned spectral peaks by using the 'ComparativeMarkerSelection' module [29]. Hierarchical clustering [30] utilised Spearman's rank correlation with pairwise complete linkage. Weighted voting and *k*-nearest neighbours (*k*-NN) classifiers [31] were used to generate predictive models using spectral data from normal and pre-operative colorectal cancer sera as a 'training' set which were then applied to the post-operative sera spectra as an independent 'test' dataset. Alternatively, the post-operative sera were classified as either cancer or normal by using the predictive algorithms in an iterative, 'leave-one-out cross validation' mode [32].

## Results

**Figure 1:**
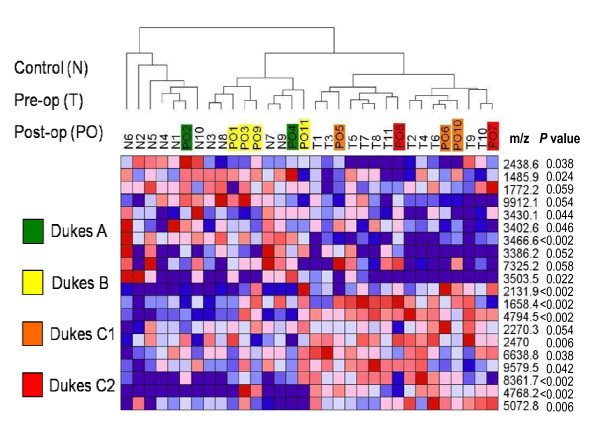
**Classification of spectra from post-operative sera by hierarchical cluster analysis**. The top 20 ranked protein peaks that discriminate normal from pre-operative CRC sera were used in hierarchical cluster analysis employing Spearman's rank correlation as column distance measure with pair-wise complete-linkage as the clustering method. The identities of serum specimens (see Table 1) are depicted in the dendrogram (N = normal, T = pre-operative cancer, PO = post-operative cancer). The Dukes' stage of each patient prior to surgery is depicted in the respective post-operative (PO) sample. The m/z values and *P *values (student's t-test) of discriminating peaks are shown in the right-hand column.

The raw spectral data used for analysis of all 32 serum samples is shown in Additional file 1. A set of 20 top-ranked marker peaks that discriminate between sera from the pre-operative cancer and the normal control groups was used for hierarchical cluster analysis (Figure 1). Six of the post-operative sera samples that belonged to early Dukes' stages A and B (PO1- PO4, PO9, PO11) were grouped with the normal controls (N) while the remaining five post-operative sera from more advanced Dukes' stages C1 and C2 (PO5-PO8, PO10) were grouped with pre-operative cancers (T) (Figure 1).

**Figure 2:**
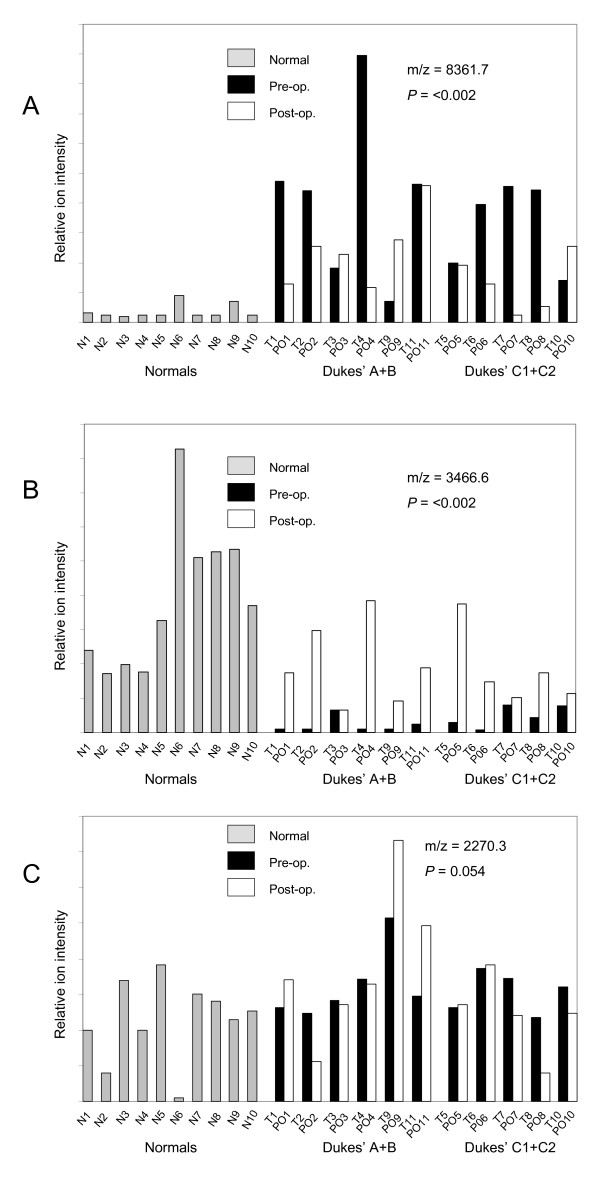
**Representative expression profiles of discriminating peaks**. The expression levels (relative ion intensity - arbitrary units) is shown for three marker peaks that discriminate between pre-operative CRC and normal control sera. The pair-wise profiles of pre-and post-operative sera are grouped according to early Dukes' (A, B) or advanced Dukes' (C1, C2) stage. The m/z and level of significance (student's t-test) for discrimination between pre-operative and normal sera is shown for each peak profile in A, B and C.

Figure 2 illustrates how the relative intensities of some representative discriminating marker peaks change in post-operative sera. The 8361.7 Da peak which is up-regulated in pre-operative CRC sera showed a marked down-regulation in most patients following surgery (Figure 2A). The 3466.6Da peak which is down-regulated in pre-operative CRC sera compared to normals was variably up-regulated in post-operative CRC sera (Figure 2B). By contrast, the 2270.3Da peak showed only borderline changes in post-operative compared to pre-operative sera (Figure 2C).

Spectra from the 10 normal controls and 11 pre-operative cancer sera were then used as a training dataset to generate predictive models for assigning an unknown serum spectrum to either the normal or cancer group. When applied to the post-operative sera spectra as an independent 'test dataset', both the Weighted voting and *k*-NN classifiers assigned the post-operative sera from the two Dukes' stage A patients (PO2, PO4) and two of the four Dukes' stage B patients (PO1, PO3) to the normal group, while the remaining two Dukes' stage B post-operative sera (PO9, PO11) were assigned to the cancer group (Table 2). All post-operative sera from more advanced Dukes' stage patients were classified as belonging to the cancer group. Identical results were obtained when the predictive algorithms were employed in 'leave-one-out' cross-validation mode to classify the post-operative sera spectra (Table 2). The classifier algorithms thus discriminate between post-operative sera from early (Dukes' A, B) and advanced (Dukes' C1, C2) stage disease (*P *= 0.045; Fisher's exact test).

**Table 2:** Classification of post-operative sera by supervised learning

Patient sera Post-op	Dukes'/TNM stage	Classification using tumour *verses *normal model				Classification using leave-one-out x-validation			
		
		*k*-NN	Conf.	WV	Conf.	*k*-NN	Conf.	WV	Conf.
PO1	B (pT3)	N	0.3828	N	0.4044	N	0.0723	N	0.3997

PO2	A (pT2)	N	0.6741	N	0.3174	N	0.2472	N	0.0249

PO3	B (pT3)	N	0.0891	N	0.0448	N	0.2052	N	0.1

PO4	A (pT2)	N	0.3015	N	0.8506	N	0.4608	N	0.2614

PO5	C1 (pT3, pN1)	T	0.11	T	0.2675	T	0.1291	T	0.1098

PO6	C1 (pT4, N1)	T	0.418	T	0.3068	T	0.5647	T	0.4638

PO7	C2 (pT2, pN2)	T	0.114	T	1.0	T	0.1973	T	0.1213

PO8	C2 (pT4, N1)	T	0.7767	T	0.572	T	0.0559	T	0.1747

PO9	B (pT4)	T	0.6758	T	0.7736	T	0.5172	T	0.0306

PO10	C1 (pT3, pN2)	T	0.234	T	0.2663	T	0.4345	T	0.5141

PO11	B (pT4)	T	0.8295	T	0.1734	T	0.5647	T	0.0475

The classification of each post-operative (PO) serum sample as being either normal (N) or cancer (T) is shown together with the confidence value (conf.) representing the proportion of 'votes' assigned to the predicted class [25]. The weighted voting (WV) and *k*-nearest-neighbours (*k*-NN) algorithms were used to classify PO samples either by first generating a predictive model from a training set comprised of normal and pre-operative cancer sera or else by 'leave-one-out' cross validation using the complete set of spectra. The feature selection statistics used for both algorithms was SNR; distance measure between each feature for the *k*-NN algorithm was Euclidean.

## Discussion

Analysis of the low molecular weight 'peptidome' in circulating blood serum/plasma by mass spectrometry protein profiling, using either MALDI-TOF MS or SELDI-TOF MS platforms, has attracted considerable interest as an approach for diagnosis of common types of cancer [14,18,32,33]. Few studies have sought to identify examples of individual diagnostic peptides in the serum of cancer patients from the burgeoning number reported by different laboratories. However, from the limited data available, such diagnostic peptidome fragments appear to largely comprise a collection of 'surrogate' tumour markers that arise from the complex interaction between the tumour microenvironment and the circulatory system [18]. Although the reliability/reproducibility of serum protein profiling by mass spectrometry has been the subject of much debate, most authorities acknowledge its potential as a diagnostic aid provided that certain safeguards are met [34]. In our study, we followed a standardised regimen of sample collection, storage and processing in order to minimise pre-analytical factors as a source of between-sample variation.

Some earlier studies of rectal and colorectal cancer have suggested that serum proteomics profiling may also yield prognostic information, in addition to potentially predicting response to treatment. For example, the changes in serum peptidome profile that occur 1-2 days following neo-adjuvant therapy in rectal cancer patients reportedly discriminate good from poor responders with 87.5% sensitivity and 80% specificity [35]. An additional study has also reported that some minor post-operative changes in the serum peptidome profile of colorectal cancer patients do occur at 2 weeks following surgery, although their relation to disease status has not been addressed [36].

In our own studies, we have investigated the application of serum protein profiling by MALDI-TOF MS for monitoring colorectal cancer patients following surgery with a view to exploring the future potential value of this approach for guiding individualised adjuvant chemotherapy. To be clinically useful, 'informative' post-operative changes would need to be evident prior to the commencement of adjuvant chemotherapy (nominally, around 6 weeks post-surgery). Therefore, an important goal of our study was to establish whether significant changes in post-operative sera could be detected at this time. We found that 4 out of the 11 patients' post-operative sera were classified as having reverted to normal when using prediction classification models that had been built using the pre-operative cancer sera and normal control sera profiles as a training dataset. Significantly, all four of these patients were early Dukes' stage (two stage A, two stage B). Post-operative sera from an additional two Dukes' stage B patients together with those of all Dukes' C1 and C2 patients were classified with the pre-operative cancer group. Broadly similar results were obtained using the less stringent hierarchical clustering classification method, except that all four Dukes' stage B, together with the two Dukes' stage A post-operative sera were classified as normal.

Our findings that the diagnostic peptidome signatures of post-operative sera from early Dukes' stage patients appear to largely revert to a more normal profile whereas those from more advanced Dukes' stage patients still retain predominant characteristics of cancer patients is in accordance with the expected clinical outcome of the patient groups; disease-free survival would be expected for most Dukes' stage A and around half of Dukes' stage B patients, while more advanced Dukes' stage patients are more likely to succumb to disease recurrence [1,2,5-8]. However, caution should be exercised in interpreting the findings from our pilot study since the sample size was small and the predictive model, which was based on pre-operative sera from the same patient cohort, utilised a low sample to feature ratio with the attendant likelihood of 'overfitting'. On the other hand, the post-operative spectral dataset was analysed 'blind' and information on disease stage of patients was not factored in to the predictive model. Nevertheless, in order to assess its clinical relevance, this approach needs to be evaluated on a much larger patient group and using an independent cohort of post-operative sera. Our findings should therefore be viewed only as a pilot methodological study.

Future larger-scale studies may well adopt a different strategy and would likely be focussed on a smaller number of informative protein peaks. It would also be highly desirable to establish the identities of discriminating protein peaks. This might facilitate the use of a more accurate/reliable quantitation method than that offered by mass spectral measurements of relative ion intensity. Finally, a larger-scale study should minimise possible confounding variables unrelated to cancer status that may spuriously affect protein peak abundance in repeated sampling from the same patient.

Further prospective studies of a larger patient group will also be needed to evaluate whether this approach could be used to inform clinical decisions on adjuvant chemotherapy, particularly for Dukes' stage B patients for whom current pathological staging of tumours is inadequate. Longer-term follow-up of post-operative sera by this approach might also offer the prospect of more reliable detection of the onset of disease recurrence than currently offered by carcinoembryonic antigen (CEA) [37].

## Conclusions

Our preliminary pilot methodological study establishes the proof-of-concept of using mass spectrometry-based serum proteomic profiling for monitoring CRC patients following surgery.

## Competing interests

The authors declare that they have no competing interests.

## Authors' contributions

CCLL collated specimens and patient data, performed some laboratory work, data analysis and drafted the manuscript. AM performed laboratory work and data analysis. NJW contributed to collation of specimens and patient data and laboratory work. SM and TA contributed to the study design, interpretation and drafting of the manuscript. JDN contributed to the study design, mass spectrometry analysis, data analysis, laboratory work and drafting of the manuscript.

## Acknowledgements

The authors acknowledge the financial support of the Royal College of Surgeons of England for this work and the contribution of Mr James Wright in preliminary experiments.

## Supplementary Material

Additional file 1 **Raw MALTI-TOF mass spectral data of all 32 serum samples used in the study**. Peak ion intensity is shown on the y axis; the x axis shows the m/z scale. Samples within each group are arranged from right to left in numerical order. Click here for file
