# Supplementary material for: Analysis of post-operative changes in serum protein expression profiles from colorectal cancer patients by MALDI-TOF mass spectrometry: a pilot methodological study
Source: World J Surg Oncol. 2010 Apr 26;8:33. doi: 10.1186/1477-7819-8-33 (PMC2873338; doi:10.1186/1477-7819-8-33)

Additional file 1: Raw MALDI-TOF mass spectral data of all 32 serum samples used in the study. Peak ion intensity is shown on the y axis; the x axis shows the m/z scale. Samples within each group are arranged from right to left in numerical order.

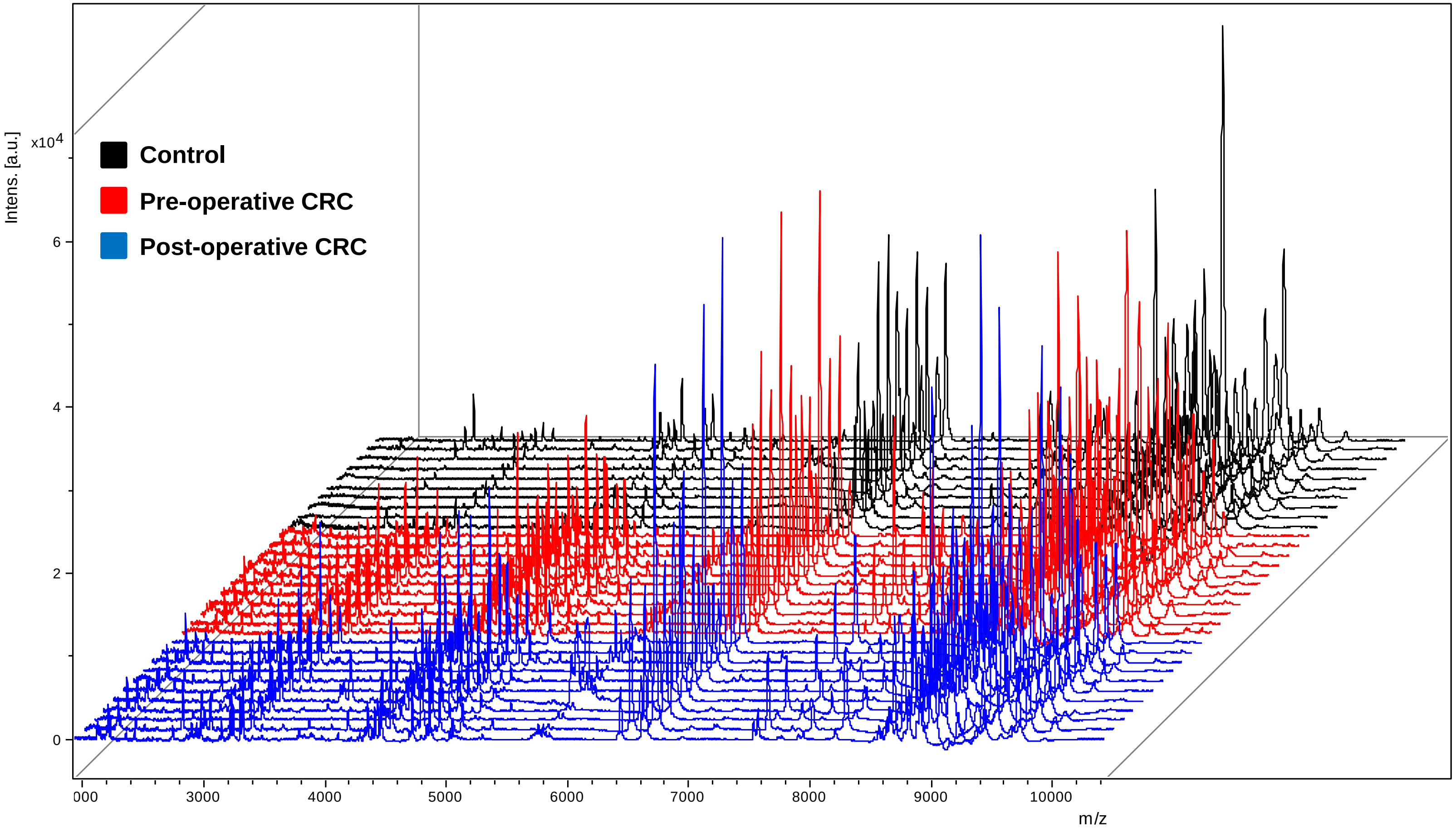

Supplement: Additional file 1 — Raw MALTI-TOF mass spectral data of all 32 serum samples used in the study. Peak ion intensity is shown on the y axis; the x axis shows the m/z scale. Samples within each group are arranged from right to left in numerical order. [file 1477-7819-8-33-S1.PDF]
